# Increasing MRI capacity at a clinical diagnostic centre and a trauma hospital using artificial intelligence-based image reconstruction (AI-IR): a quality improvement project using the Model for Improvement framework

**DOI:** 10.1136/bmjoq-2025-003470

**Published:** 2025-11-04

**Authors:** Joe Martin, Zohreh Hurcum, Susan Cross, Francisco Pepito Ablen, Sunitha Sivarajah, Marianthi Vasiliki Papoutsaki, David Adams, Agnieszka M Peplinski, Rosy Jalan, Krishanantham Ambalawaner, Rozeta Bennett, Sujit Vaidya, Dina Pefanis, Sara Moeen, Sivadas Ganeshalingham, Muaaze Ahmad, Hannah Dupreez, Nathan Proudlove, Marc Eric Miquel

**Affiliations:** 1 Barts Health NHS Trust, London, UK; 2 The University of Manchester, Manchester, UK; 3 Alliance Manchester Business School, The University of Manchester, Manchester, UK; 4 Guy's and St Thomas’ NHS Foundation Trust, London, England, UK; 5 King’s College London School of Biomedical Engineering & Imaging Sciences, London, England, UK

**Keywords:** Diagnostic Imaging, Artificial Intelligence, PDSA

## Abstract

Increasing MRI capacity is of primary importance to both NHS England and individual radiology departments. Consequently, central funding was provided to allow trusts to instal artificial intelligence-enabled image reconstruction (AI-IR) on their MRI scanners, with the stated aim of increasing capacity by two patients scanned per day within a year of installation on a given scanner. This work demonstrates how a two-phase quality improvement (QI) initiative can be followed to increase capacity using AI-IR in a community diagnostic centre (CDC) at Mile End Hospital and an acute trauma centre, the Royal London Hospital, in East London with comprehensive stakeholders’ engagement.

The Model for Improvement framework was used. Our pilot study focused on 3 Plan-Do-Study-Act (PDSA) cycles for three anatomies in musculoskeletal (MSK) imaging at our CDC. A second, substantive study at our major trauma centre was followed, which was a 20-month project encompassing all MSK anatomies of interest.

In our initial pilot study at the CDC, we were able to reduce booking times by 10 min for Knee, Ankle and Spine protocols. In our wide-ranging MSK programme at our trauma centre, we saved on average of 07:26 min per scan and while an increased throughput was not achieved, an increase in complex patients being scanned, from 7% to 15% was achieved, reducing healthcare inequities.

Our two-centre study suggests that engaging with stakeholders in a structured QI programme can significantly reduce scanning times, improve patient experience and allow for longer precare and postcare time. Additionally, significant throughput increase at the CDC for low-risk ambulatory patients suggests efforts to increase capacity using this technology should be focused at such centres and other scanners focused on ambulatory outpatients, while for scanners focused on inpatients, paediatrics and A&E at trauma centres, the time saved can be used to increase the capacity for complex patients, reducing waiting times for these patients.

**WHAT IS ALREADY KNOWN ON THIS TOPIC**

- Artificial intelligence (AI) reconstruction in MRI is an exciting new development with the potential to greatly quicken MRI scanning, increasing capacity while improving patient comfort. However, this technology is highly novel and its efficacy for our local patient population is unknown.

**WHAT THIS STUDY ADDS**

- This work is the first description of a quality improvement project to validate and implement AI reconstruction in MRI for musculoskeletal maladies in a highly diverse patient cohort.

**HOW THIS STUDY MIGHT AFFECT RESEARCH, PRACTICE OR POLICY**

- This methodology, when duplicated, can be used to successfully used to validate AI in MRI for quicker scanning while ensuring accurate imaging for our patients, particularly for MRI scans of ambulatory outpatients.

## Problem

Since clinical MRI began, there has been a continual rise in the number of patients referred for scans; in the last 30 years, this increase has been exponential. The 2019 *NHS Long Term Plan*^1^ highlighted the need for expanded MRI capacity due to ever-increasing referral numbers. Subsequently, *NHS England’s* post-pandemic Diagnostics: Recovery and Renewal plan^2^ further highlighted the need for more scanners, coupled with the need to maximise capacity on new and existing equipment, while also justifying the creation of Community Diagnostic Centres (CDCs), aimed at routine outpatient scans including MRI. Concurrently, *NHS England,* after assessing available options,^3^ decided to centrally fund artificial intelligence-enabled image reconstruction (AI-IR) in MRI, with the stated aim of adding two scans per day after a year of implementation.

Our Trust, Barts Health, was successfully applied for this funding, and a pilot quality improvement (QI) programme to implement it was trialled over 5 months at our CDC based on the Model for Improvement (MFI). Building on this work, a subsequent comprehensive QI project for all our musculoskeletal (MSK) MRI imaging at our major trauma centre was conducted over 20 months. The primary aim was to reduce scanning times and thus increase the number of patients scanned by two per day; which is the stated objective of the central funding. However, while the CDC almost entirely scans ambulatory outpatients, the trauma centre scans many highly complex inpatients (including sedated and general anaesthetic (GA) patients), patients with active implanted devices, Accident & Emergency (A&E) and multiple trauma patients and many paediatric and neonatal patients. These complexities will intrinsically add to patient set-up time, as radiographers must invest significant time to ensure the safe scanning of these patients. Consequently, although scanning time can be reduced, the overall reduction in patient booking time is intrinsically limited by those factors.

This study offers a case study in implementing the novel AI-IR technology into clinical use, with the primary aim of increasing capacity. However, as well as benefiting the patient population by offering potentially quicker access to imaging, and thus potentially quicker diagnosis or treatment follow-up, individual patients benefit from the reduced time on the scanner. MRI is a noisy, enclosed environment where patients often must lay still on their backs on an uncomfortable flat bed. This is not well tolerated even by healthy volunteers, and patients with conditions such as back pain, other MSK maladies, claustrophobia, various forms of neurodiversity and paediatric patients often find this extremely difficult. This often leads to failure to achieve diagnostic-quality imaging and ultimately a healthcare inequity. Image artefacts are image features created erroneously that cause a reduction in image quality or can cause features to appear that do not correspond to the patient’s anatomy, such as artefacts caused by patient motion, which can lead to misdiagnosis. Concurrently, quicker scanning reduces motion artefacts, which again benefits the patient.

This paper describes both the QI project design and initial PDSA cycles at the CDC and at the major trauma centre for MSK imaging. This work, spread via regional and national groups, will aid other centres who are contemporaneously attempting to implement this technology in clinical practice.

This MFI-based QI initiative primarily focuses on a project to implement AI-IR in MRI, first in a pilot at an outpatient CDC and then a more substantial test at a major trauma centre in East London with the aim of increasing MRI capacity on a *Siemens VIDA* and *Sola* MRI Scanners using their AI implementation, *Deep Resolve*. Other parallel and concurrent processes also aimed at improving capacity are discussed, to contextualise this QI process.

## Background

As stated above, the need for increased MRI scanning capacity is a paramount concern for both NHS England and our Trust locally. The number of patients scanned on a particular day at a given centre is ultimately determined by:

- Number of MRI scanners.
- Opening hours.
- Length and number of MRI sequences and how well optimised they are.
- Complexity of scans.
- Patient’s mental and physical condition.
- Patient’s implanted or accompanying medical devices.
- Available radiographers.
- Number of ‘did not arrives’ (DNAs) and patients unable to be scanned for safety reasons.

Additionally, a driver diagram^4 5^ for increasing capacity on MRI can be viewed in online supplemental material S1. Optimisation is the process of improving the imaging sequences, and creating new ones, for example, to give better diagnostic information, or reduce scan time. This is often led by an MRI physicist as an expert on sequence theory and radiographers, who are experts on anatomy and image acquisition, with radiologists giving feedback on the resultant images. Other QI projects to improve MRI capacity have focused on methods such as improving patient throughput efficiency,^6^ lean principles,^7^,^9^ smart booking^10 11^ and standard protocol optimisation,^7 12^ but we believe, following a thorough literature review, this is the first to assess the benefit of AI-MRI image reconstruction on increasing capacity.

The CDC scanner is a *Siemens Vida 3T* and is used for ambulatory outpatients, operating 8am-8pm 7 days a week. The trauma centre’s *Siemens Vida 3T* and *Siemens Sola 1.5T* scanners are often used for complex patients including, *but not limited to*, adult and paediatric intensive care patients, A&E, psychiatric ward, paediatrics, neonates, other inpatients and those with active medical devices. The trauma centre’s third scanner, a *Siemens Avanto^Fit^ 1.5T scanner,* which cannot use the AI-IR software, is only used as an ambulatory outpatient scanner. The length of time available on these scanners is 12 hour days, 5 days a week at this centre, with one or two scanners open at the weekend from 9:00 to 17:00 on a voluntary-staff-overtime basis, when mainly non-complex patients are scanned together with any emergency complex patients. There is not enough radiographer capacity to increase this any further at present, particularly as the majority of radiographers are rotational and must also cover CT and X-ray. There is an active recruitment campaign for dedicated MRI staff and staff training, including for advanced techniques such as AI-IR, is also ongoing with future sessions planned to help non-clinical specialist radiographers gain the skills to optimise sequences with physicists (to be tested in future PDSA cycles). The aim is to have full capacity scanning being 7 days a week, 8:00–20:00 within the next year. Reducing outsourcing of workload is also a key driver of these initiatives; as it takes money outside of the NHS, and as they only take relatively simple scans for ambulatory patients, it burdens heavily overstretched MRI departments with a more severe case-mix and can lead to a drop in morale and retention.

Less time per patient allows increased capacity, hence the aim set by *NHS England* to add two scans a day using the AI-IR technology, but freed-up time can also be used to aid safety checks for complex patients, for example, paediatrics and GA adults. The software was installed at our centre and was ready for implementation in February 2022 at the CDC and in May 2023 for the trauma centre.

In normal operation, radiographers and physicists are constantly working to improve protocols and reducing scanning times, in consultation with radiologists, and this process has continued informally since the scanners were installed. Although other methods of image acceleration exist, known as parallel imaging, iterative reconstruction and compressed sense (CS), the consensus among stakeholders was that time savings offered by these methods had been maximised at the two centres by November 2022, with further attempts to lower scanning times leading to an unacceptable drop in image quality. AI-IR in MRI has been shown to be better than these other acceleration techniques in terms of reducing ghosting artefacts and reducing motion artefacts and outscores CS using quantitative measures such as signal-to-noise ratio and structural similarity matrix. However, large concerns remain around commercial implementations acting as *black boxes*; their models and training data are not publicly available, and this raises the concern that no symptomatic patient data were included in the training images, and about the diversity of the data in terms of protected and unprotected patient characteristics used to train the algorithms. Thus, for certain cohorts of our patients, the AI software may not work effectively and/or may reinforce existing healthcare inequalities. Consequently, a clinical validation programme is necessary.

Invoking Dodds’ Three Wins,^13^ we have considered the benefits of implementing AI-IR to reduce scanning time and resultantly increasing capacity for the three stakeholder groups: patients, staff and our Trust (see table 1), together with some risks to the project associated with each.

**Table 1:** Dodds’ three wins (and risks) for the implementation of artificial intelligence image reconstruction in MRI: potential benefits and risks (with mitigations)

Cohort	Benefits	Risks (mitigations)
Patients	Less time on scanner (particularly for those less tolerant and/or in pain). Reduced waiting time for diagnostics, leading to quicker prognosis and treatment, improving morbidity and mortality. Reduced motion artefacts leading to improved image quality and fewer recalls.	Potential increased risk of misdiagnosis? (validation procedure) Public may reject use of AI due to widespread societal concerns. 14 (PPI (Patient and Public Involvement) outreach work) Scan time reduction, but trauma centre has many patients with complex needs that limits the minimum appointment time. (no mitigation at present)
Staff	Radiographers: potentially more time for after care, increased skill level and Continuing Professional Development (CPD). Radiologists: patients receiving more timely scans and fewer artefacts reduces reporting pressure and reduce the need to request patient recalls.	Physicists and radiographers incorrectly implement the software leading to erroneous imaging and misdiagnosis. (validation procedure and working with national and international colleagues) Radiologist mistrust of AI hinders adoption. (initial discussions with stakeholders and validation procedure, in addition to national stakeholder engagement)
NHS Trust	Increased revenue for radiology departments. Reduction in outsourcing (and money spent outside the trust) Reduction in breaches (and fines), including for climate-impact issues. Timelier interventions require fewer radical treatments (and thus are cheaper). Reduction in healthcare inequalities (as required by the National Health Service Constitution).	Reputational risk if project not implemented successfully. (adequate resources to staff implementing) Litigation risk from misdiagnose. (validation procedure) Initial drop in capacity due to image duplication, causes further patient delays and breach fines. (design of QI programme, see below)

AI, artificial intelligence; QI, quality improvement.

However, there is a finite minimum scanning time for patient appointments, due to patient set-up time and aftercare. This includes checking they have no metal about their person before they enter the room, helping them onto the scanner bed (requiring a pat-slide patient transfer board for non-ambulatory and GA patients), placing the receiver coils on them and patient alignment. Post-scan, this process is reversed along with patient aftercare and cleaning the bed (with increased infection control processes in place since the pandemic). Complex patients, as defined above, require more set up time and aftercare.

## Measurement

In an MRI session, each patient will have a number of different scans showing different image contrasts and information; collectively, these are known as *‘scanning protocols’*. With a focus on optimising protocols to reduce scan time using AI-IR in MRI, the metrics were:

Time saved per protocol - a minimum of 5 min will be required to change a booking time. – the baseline protocol times are found in column 1 for each protocol chosen/PDSA cycle in online supplemental material S2.

The overarching aim is an increase in capacity by at least 2 patients per day after a year, and thus the next metric is:

Weekly average of number of scans per day – Baseline measurement: the average for the first 3 months of opening prior to AI installation is 8.

## Design

Our methodology utilised the MFI framework and PDSA cycles. The MFI consists of 3 systems questions to establish aim, metrics and initial change ideas, followed by PDSA cycles to test and refine these ideas. It has been used in many NHS clinical sciences QI projects,^14^,^16^ including in medical physics^17^ and in our Trust.

### Initial pilot study: CDC

The core implementation team was led by MRI physicist for the two centres (lead author) and also comprised the Clinical Specialist Radiographers at each centre (FPA, ZH). We had attended applications training with the manufacturers’ representatives, who taught us how to use the Siemens’ Deep Resolve software,^18^ indicating its primary application was for sequences using “Turbo Spin Echo” in addition to it being highly successful for MSK imaging. In addition to our work at the CDC, we spoke to the head of MSK radiology at the trauma centre the CDC is attached to, but we found engagement from their radiologists minimal, likely due to no MSK radiologists being embedded at this site. We thus relied on ourselves to view the imaging and the knowledge that if image quality worsened, we would then get reactive feedback from the radiologists.

### Substantive study: major trauma centre

**Figure 1:**
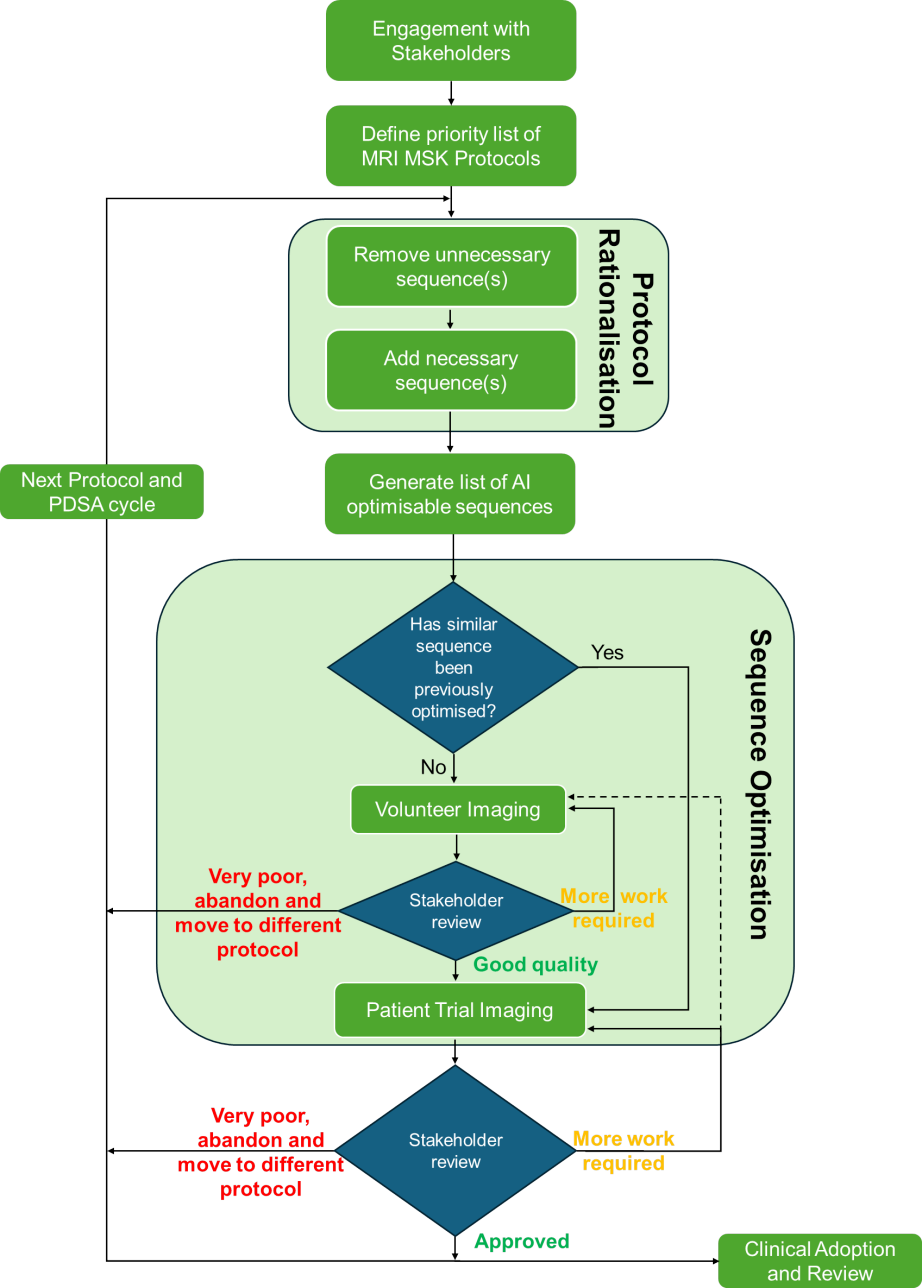
Flowchart demonstrating the process used for the quality improvement (QI) programme for reducing scanning time using AI-IR in MRI for multiple imaging protocol and anatomies of interest in MSK at our major trauma centre. AI-IR, artificial intelligence image reconstruction; MSK, musculoskeletal; PDSA, Plan-do-study-act.

Following on from the initial trial study, and lessons learnt as described above, we performed PDSA cycles at our trauma centre utilising the method in figure 1. When we began here, physics spoke to the hospital’s head of MSK radiology, multiple consultant radiologists and other MSK radiologists, as well as a broader cohort of radiographers, and explained the purported process. As stated above, due to the limit in feedback at the CDC, which we believed was in large part due to no embedded specialist radiologists at the centre, we formed an MDT at the trauma centre, which consisted of the MR physicists, two specialist radiographers and 4–5 senior MSK radiologists. MRI image optimisation projects, when looking at a single or small number of sequences, typically use questionnaires and scoring metrics, such as Likert,^19 20^ to compare preoptimisation and postoptimisation images, ideally blinded. This often takes multiple months to perform with radiologists for a single MRI sequence, and as this project was intended to optimise over 100 sequences, this was not practicable. Therefore, the radiologists gave a consensus binary response, ‘is it diagnostic and at least of equal standard to previous imaging or needs improvement?’, and if the latter, they also provided an explanation of their requirements, before the start of a further PDSA cycle to improve diagnostic quality.

Consequently, a priority list for protocol development was created based on the radiologists’ recommendations, with weight also placed on the frequency of imaging. Consideration was also given to where optimised sequences could be translated to other anatomy of interest and easily modified for another with similar tissue composition, for example, knee to ankle, elbow to shoulder. The testing for each chosen protocol was:

- Volunteer sessions with physicist and radiographers to initially test and request feedback.
- Further optimisation on patients. This initially means that there is an initial reduction in scanning capacity during each cycle, although different cycles for different anatomies and sequences were running concurrently.
- Implementation and review at 1, 3 and 6 months.

Multiple protocols would go through this process concurrently, dependent on capacity to conduct scanning on the volunteers, which also will reduce clinical capacity, but was limited to two or three as we found that to be a manageable number.

## Strategy

### CDC Pilot

A summary of the three completed PDSA cycles from the pilot scheme at the CDC is seen in online supplemental material S2.

For the first PDSA cycle, the knee protocol was chosen as it had five sequences where AI-IR could be applied. Initially, a 5 min reduction was achieved, and this was tested on multiple patients. We asked for direct feedback from radiologists. The only feedback received stated they were ‘fine’ in comparison to existing clinical sequences, so due to time saving, we chose to adopt this sequence, and it has subsequently been audited as successful for a wide cohort of our patients.

The second PDSA cycle, to develop an ankle protocol, utilised anatomical similarities to the knee, so drew on work from the first cycle, which allowed for quicker iteration and improvement. For this reason, optimisation moved straight to patient imaging, as volunteer scans were not necessary due to the similarity. In this case, requests for feedback from radiologists went unanswered, but checking reporting of images showed no complaints were received or additional recalls were requested. As both myself and the clinical specialist radiographer were pleased with the imaging, we made the decision to adopt the new clinical protocol and enter the review phase. This protocol was further optimised with radiologist feedback in further PDSA cycles as part of our substantive work at the trauma centre (see below).

The third cycle focused on spinal imaging, selected due to the large number of such cases at our centre. We first optimised the lumbar spine protocol and after success, was adapted for the different anatomies of the cervical spine and thoracic spine. Once more, feedback was limited for these anatomies; however, when we attempted to adapt these gains for whole-spine imaging, both ourselves and the radiologist noted novel-looking artefacts that could lead to misdiagnosis (online supplemental material S3). Working with Siemens, we corrected this issue and have successfully updated all spine protocols with significant time reductions, allowing for a 10 min reduction in booking times.

### Trauma centre MSK programme

Following on from the initial trial study, and lessons learnt as described above, we performed PDSA cycles for each protocol at our trauma centre utilising the method in figure 1. This work covered the ankle, knee, head, thumb, hip, pelvis, fingers, wrist, elbow, shoulder and spine protocols, and the radiologists approved each quickened, optimised protocol to be of equal or improved image quality, in addition to time saved.

## Results

### CDC Pilot

**Figure 2:**
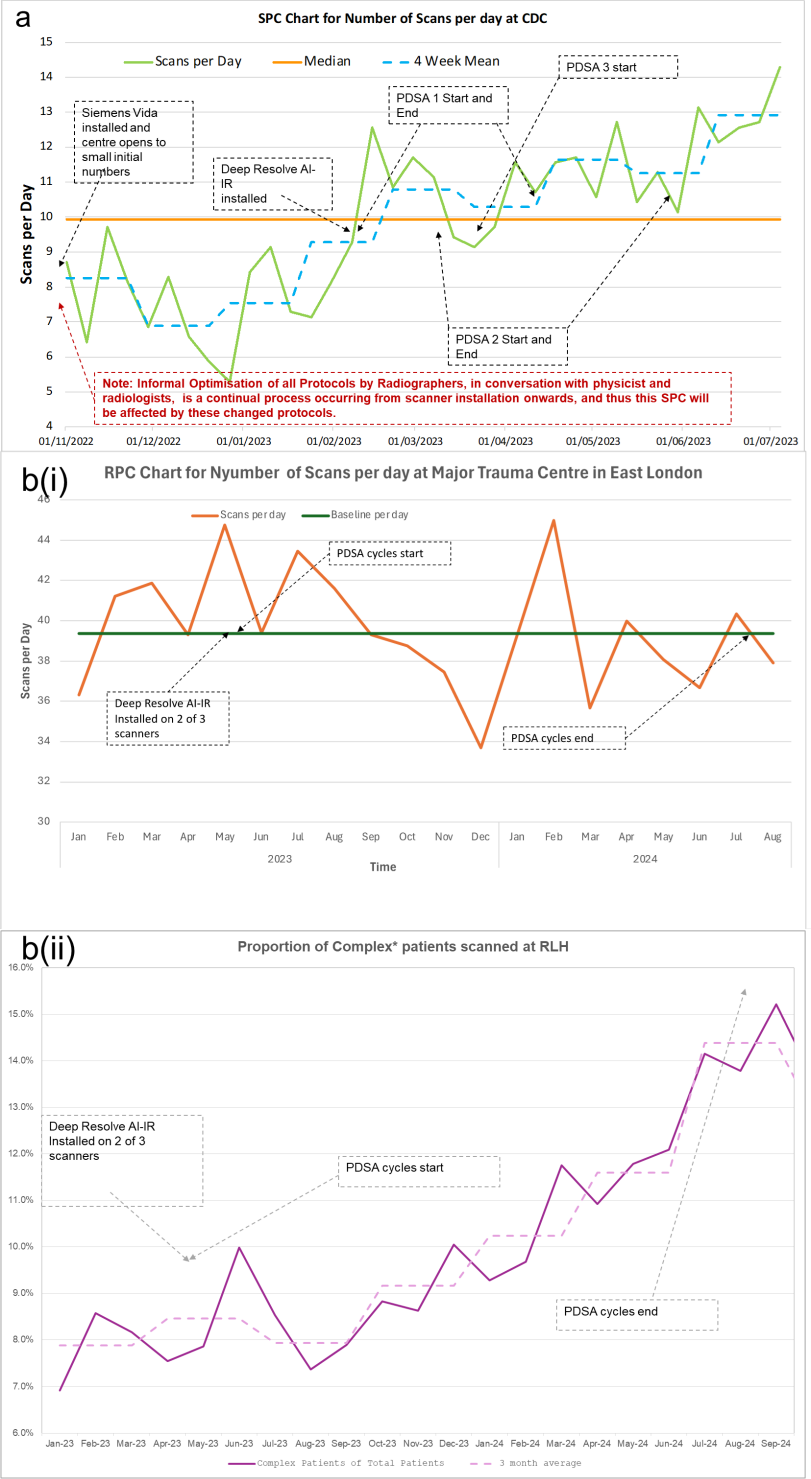
QI in MSK at a CDC (a) and a trauma centre (b): (a) run chart for CDC MRI average scans per day before and after the implementation of AI in MR reconstruction. (b) (i) Run chart for trauma centre MRI scans per day before and after the implementation of AI on 2 of its 3 scanners. (b) (ii) Percentage of complex patients scanned at our trauma centre. This includes but is not limited to adult and paediatric intensive care patients, A&E, psychiatric ward, paediatrics, neonates but not patients with complex medical implants. AI-IR, artificial intelligence-image reconstruction; CDC, community diagnostic centre; QI, quality improvement; MSK, musculoskeletal; PDSA, Plan-do-study-act; A&E, Accident & Emergency.

The run chart (figure 2a) shows increased capacity since the installation of AI-IR (*Siemens Deep Resolve*) in February 2022. Ultimately, *NHS England* aimed for two extra scans per day within a year of installation; at our centre, we are achieving an average of four more per day after 5 months and six more per day by the end of the trauma centre PDSAs, whose finalised shorter MRI sequences were transferred to the CDC where applicable.

### Trauma centre MSK

In our wide-ranging MSK QI programme at our trauma centre, the mean timesaving achieved was 07:26 (min:s) (range: 2:28 to 19:15) per patient. The full results and time savings are found in online supplemental material S4.

Despite the time savings achieved, the number of scans per day at our trauma centre has remained approximately static (figure 2b). However, the proportion of complex scans performed at this centre increased during the PDSA cycles from 7% initially to 15% at the end (figure 2c), and this is an underestimate as complex-device patient data were unavailable.

This suggests to us that for scanners focused on ambulatory inpatients (such as at our CDC), this AI software can successfully increase the patient throughput, while on scanners focused on inpatients (such as at our trauma centre), the scanning time reduction is best used to increase inpatient and complex patient access, reducing healthcare inequities, ensuring they are scanned safely and with appropriate before and after care.

## Lessons and limitations

As noted above, this project did not occur in a vacuum. Imaging protocols develop over time and are constantly optimised informally, and so the increase in number of patients scanned might not be solely due to our QI project. This work focused on MSK imaging, due to the frequency of scanning and number of available sequences that the software can be used on as well as the consequences to patients from non-diagnostic (poorer quality) images or misdiagnosis being less severe, with extremely low increased risk of mortality. Recently, we have had Siemens Deep Resolve for other types of scans installed, HASTE^21^,^23^ which is highly useful for abdominal scanning and diffusion-weighted imaging, which is particularly useful for stroke and cancer imaging. We will use these for future QI projects for neurological and abdominal imaging, which forms a significant amount of our scanning requests at both centres, and cancer imaging in general, as we now have more confidence in the software, thanks to the initial pilot and MSK PDSA cycles. On reflection, in future cycles, we will monitor the inpatient waiting time, which we did not monitor for this work as we did not predict the increase in complex patients being scanned at our trauma centre.

Patients and the public more generally are both excited and sceptical of AI, with leading AI companies themselves suggesting it could represent an existential threat.^24 25^ To understand our patient population’s views on the use of AI-IR, we published and advertised a questionnaire (online supplemental material S5 and S6), after which we had two focus groups explaining AI-IR in a lay manner, to explain the issues, particularly around ethics and diversity of training data. In general, patients were excited but apprehensive and believed the manufacturers should publish their data and models and state the accuracy, which they currently do not.^26^ We have since expanded this work to include all stakeholders’ views, with questionnaires for radiographers, radiologists and physicists, with the aim to publish these findings in the near future.^27^

Smart booking, where same or similar scans, which require similar set up with efficient use of time slots, would allow any time reductions to be better implemented into additional patient slots. This will form part of future QI work, as discussed above, as will new patient letters intended to reduce set-up time, DNAs and patients unable to be scanned for safety-screening reasons.

Many centres have also been early adopters of this technology, so developing protocols in isolation introduces duplication waste across the NHS. To reduce this, the corresponding author has set up a *London NHS MRI AI User Group,* that is, meeting monthly and sharing knowledge and protocols. Consequently, we would expect future PDSA cycles to be quicker (potentially 1–2 months instead of 3–6 currently), and thus benefits could be realised more quickly.

## Conclusion

In this work, the MFI and its PDSA cycles were used to successfully reduce scanning times at our CDC and trauma centre and achieve an increase of six patients per day by the end of this QI project, exceeding *NHS England’s* aim of 2 extra after a year. While increased throughput was not achieved at the trauma centre, an increase in complex patients occurred, reducing healthcare inequalities. Additionally, the significant increase in throughput at the CDC for low-risk ambulatory patients suggests efforts to increase capacity using this technology should be focused at such centres and on dedicated outpatient scanners.

## Supplementary material

10.1136/bmjoq-2025-003470 online supplemental file 1

## Data Availability

No data are available.
