# Supplementary material for: Increasing MRI capacity at a clinical diagnostic centre and a trauma hospital using artificial intelligence-based image reconstruction (AI-IR): a quality improvement project using the Model for Improvement framework
Source: BMJ Open Qual. 2025 Nov 4;14(4):e003470. doi: 10.1136/bmjoq-2025-003470 (PMC12587991; doi:10.1136/bmjoq-2025-003470)
Supplement: online supplemental file 1 [file bmjoq-14-4-s001.docx]

**Supplementary Material**:

**Supplementary Material S1: Driver Diagram**

*Figure S1: Driver Diagram for Increasing imaging Capacity on MRI scanners at a CDC and a major trauma centre in East London (Barts Health Trust)*

**Supplementary Material S2: Pilot Study at CDC PDSA Cycles**

*Table S1: Summary of the first 3 PDSA cycles run at the Community Diagnostic Centre (pilot study)*

| **PDSA Cycle** | **Plan / Prediction** | **Do** | **Study** | **Act** | **Time Required** |
| --- | --- | --- | --- | --- | --- |
| 1 | Knee Scanning Optimisation.  Initial Scan Length: 17-21 mins, predict ~5 mins reduction  Initial Booking Time: 30 mins, | Test on a volunteer (the author)  Send images to radiologists | 5 min reduction achieved.  Feedback from radiologists: images were “fine” and of equivalent quality: Physicists and Radiographers happy with quality of images. | Further Optimisation on patients and then protocol changed. Review process underway.  **New Booking Time: 20 mins** | 3 months |
| 2 | Ankle Scanning Optimisation  Initial Scan Length: ??? etc  Initial Booking Time: 30 mins | Adapt sequences optimised for knee. | Added to sequence and sent to radiologists.  No feedback but no added complaints or recalls: Physicists and Radiographers happy with quality of images. | Further Optimisation on patients and then protocol changed. Review process underway.  **New Booking Time: 20 mins** | 3 months |
| 3 | Spinal Imaging –  t-spine, l-spine, c-spine  Initial Scan Length: 25-27 mins  Initial Booking Time: 30 mins | Began with l-spine.  Then adapt for c-spine and t-spine. | Added to sequence and sent to radiologists.  No feedback aside from concerns of artefacts on whole spine images (Supplementary Mat. C), which were also noted by physicist and radiographers. Further iterations occurred and with Siemens help resolved the artefacts. | New t-spine, c-spine and l-spine clinical protocols adopted..  New Booking Time: 20 mins  **New Booking Time: 20 mins** | **5 months-** |

**Supplementary Material S3: Image artefact in whole spine imaging**

**
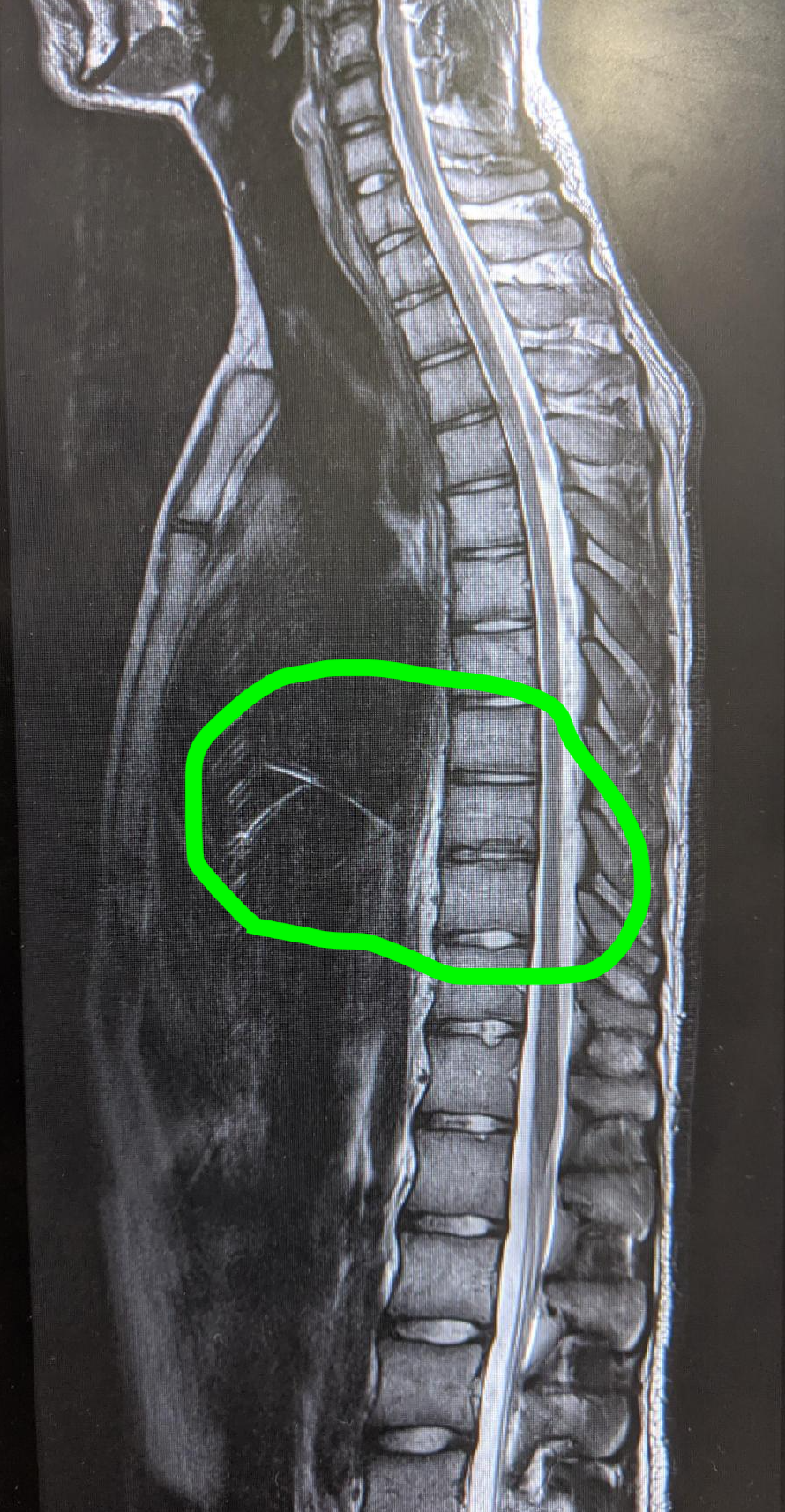
**

Figure S2: Streaking artefact in the spine when using Siemens Deep Resolve AI-IR. This was later resolved with the aid of Siemens applications specialists.

**Supplementary Material S4: Scanning Time reductions at Trauma Centre**

*Figure S3: Time Savings for different MSK MRI scanning protocols at a trauma centre in East London following an extensive QI project based on PDSA cycles and the Model for Improvement*

*Table S2****:*** *Time savings on the Siemens Sola (1.5T) and Vida(3.0T) at Royal London Hospital for MSK protocols. Each row represents a different sequence which forms part of the imaging protocol, with the whole scan times displayed at the bottom of each column. DR is the times using Deep Resolve AI-IR, where ‘original’ is the sequences without AI or sequence rationalisation.*

**Supplementary Material S5: Poster for Patient and Public Involvement and Engagement Questionairre on AI-IR.**

*
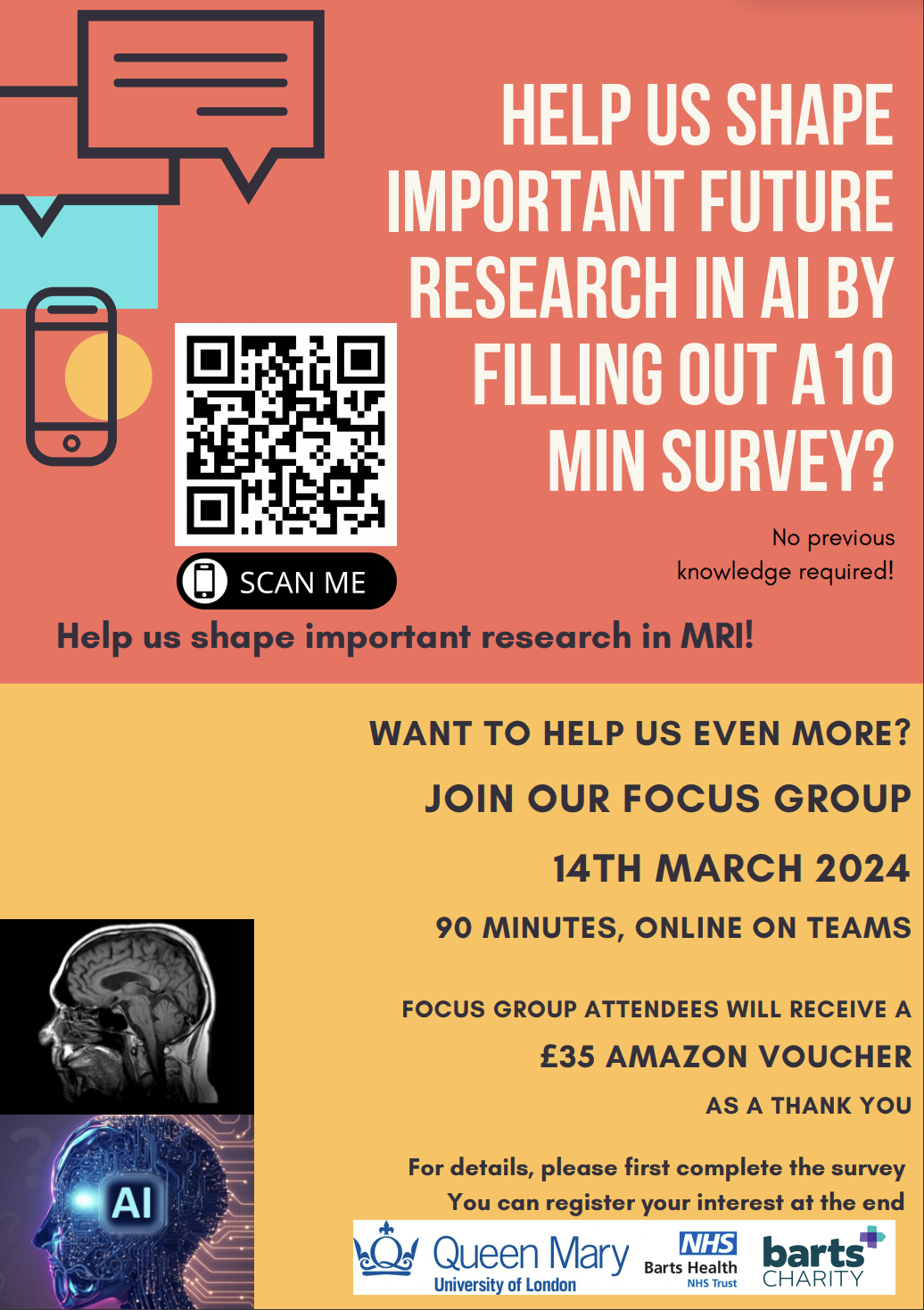
*

*Figure S4: Poster with QR code displayed in MRI waiting rooms throughout our trust, produced in collaboration with the Barts Health PPIE team.*

**Supplementary Material S6: PPIE Questionnaire Questions**

1. Have you had an MRI before? If yes, roughly how many?
2. Please describe your experience when you had an MRI
3. How familiar are you with artificial intelligence in healthcare?
4. Have you personally experienced or interacted with any healthcare services that use artificial intelligence? If yes, please describe your experience.
5. What are your initial thoughts or feelings about the use of artificial intelligence in healthcare?
6. Do you think there are any benefits to using artificial intelligence in healthcare? Please describe them.
7. Do you have any concerns? Please describe them.
8. Do you believe that artificial intelligence could help healthcare professionals make more accurate diagnoses and treatment decisions? Why or why not?
9. Do you think there are any ethical considerations or potential risks when using artificial intelligence in healthcare that you think should be addressed?
10. How comfortable would you be with the idea of a healthcare AI system assisting or advising your healthcare provider in making treatment decisions? 1-5 rating
11. Would you like to be made specifically aware if artificial intelligence technology is used in your diagnosis?
12. When you undergo an MRI scan often the longer you are scanned for, higher the quality of the image. How do you feel about having long MRI scans?
13. You can take an image in a shorter time, but you only acquire some of the information, and you end up with an image that is lower quality (e.g. more blurry). The AI technology works by taking a lower quality image in a shorter time and making an educated guess at what information is that you didn’t measure. This turns a lower quality image into a higher quality image. How do you feel about that? 1-5 rating?
14. Would you be willing to undergo an MRI scan that uses AI technology to to improve the image quality?
15. How would you feel if the use of AI in MRI reconstruction resulted in shorter scan times and reduced the need for repeat scans? 1-5 rating
16. How important is it for you to have clear explanations and transparency regarding the use of the AI technology in your MRI scans? 1-5 rating
17. Do you think research to find techniques for making imaging quicker and using artificial intelligence methods to do that would be useful? How useful? Rate 1-5
